# Supplementary material for: Contribution of socio-demographic factors in prevalence of soil-transmitted helminth infections among newly arrived laborers in Jeddah, Saudi Arabia
Source: PeerJ. 2024 Oct 8;12:e18216. doi: 10.7717/peerj.18216 (PMC11468799; doi:10.7717/peerj.18216)
Supplement: Supplemental Information 2 [file peerj-12-18216-s002.pdf]

STROBE Checklist

SECTION	ACTION
**Title and abstract**	The study's design was indicated with a commonly used term and an informative abstract provided what was done and what was found.
**Introduction**	
Background/rationale	The scientific background and rationale for the investigation being reported were explained.
Objectives	The specific objectives were stated.
**Methods**	
Study design	Key elements of study design were presented.
Setting	The setting, locations, and relevant dates and data collection were described.
Participants	Criteria and methods of selection of participants were given.
Variables	Diagnostic criteria were given.
Data sources/ measurement	Sources of data and assessment methods were described.
Bias	Not applicable
Study size	Explained how the study size was arrived at.
Quantitative variables	Explained how quantitative variables were handled in the analyses.
Statistical methods	All statistical methods and related analyses were described.
**Results**	
Participants	Numbers of individuals participated in the study was reported.
Descriptive data	Not applicable.
Outcome data	Numbers of outcome events or measures were reported.
Main results	All main results were reported.
Other analyses	Not applicable.
**Discussion**	
Key results	Key results were summarized with reference to study objectives.
Limitations	Not applicable.
Interpretation	Overall interpretations of results were given, considering objectives, multiplicity of analyses, results from previous similar studies, and other relevant evidence.
Generalisability	The findings of the study results can be applicable to other settings.
**Other information**	
Funding	Not applicable, (this research was not funded).
